# Associations between the Application of Signature Character Strengths, Health and Well-being of Health Professionals

**DOI:** 10.3389/fpsyg.2017.01307

**Published:** 2017-07-31

**Authors:** Melanie Hausler, Cornelia Strecker, Alexandra Huber, Mirjam Brenner, Thomas Höge, Stefan Höfer

**Affiliations:** ^1^Department of Medical Psychology, Medical University of Innsbruck Innsbruck, Austria; ^2^Institute of Psychology, University of Innsbruck Innsbruck, Austria

**Keywords:** signature character strengths, well-being, health, burnout, emotional exhaustion, medical students, physicians, positive psychology

## Abstract

Previous research has shown a positive relation between character strengths, well-being and health. The aim of this analysis was to identify relations between the application of signature character strengths (ASCS) at work, and well-being and health, among medical students (Study 1) and resident physicians (Study 2). We expected positive direct links between the constructs and indirect effects through emotional exhaustion. To test these hypotheses, 387 medical students in their first year and 136 resident physicians completed five scales measuring well-being, mental/physical health, character strengths, the application of their five individual signature strengths, and emotional exhaustion as an indicator of burnout. Partial correlations were examined, and mediation analyses performed. ASCS at work was positively linked with well-being and mental health but not with physical health. All links were mediated by emotional exhaustion in Study 1 and (except for mental health) also in Study 2. Future studies would therefore do well to investigate the promotion of ASCS at work of people operating in medical education and its potential in fostering well-being and preventing burnout from the outset.

## Introduction

Positive psychology is a science that deals with three domains: (1) positive subjective experiences, (2) positive traits, and (3) positive institutions (Seligman and Csikszentmihalyi, 2000). Positive experiences include well-being, optimism, and happiness. Positive traits are individual characteristics such as virtues and talents. Lastly, positive institutions, such as universities or hospitals, can support personal growth and positive experiences (Seligman and Csikszentmihalyi, 2000; Ruch, 2008). Concerning the first domain, there are many alarming studies reporting burnout (Dyrbye et al., 2008; Dyrbye et al., 2014), (dis-)satisfaction (Shanafelt et al., 2009; Lebensohn et al., 2013), reduced quality of life (West et al., 2011; Beckman et al., 2012), fatigue (West et al., 2009), and other health restrictions of medical students and practitioners. Studies showing that physicians’ satisfaction is positively associated with patient satisfaction, efficient health care, physicians’ health and negatively associated with turnover, burnout, and costs demonstrate that the physicians’ well-being matters not only for themselves, but also for their patients and the health care system in general (Brown and Gunderman, 2006). A first study (Hausler et al., 2017b) found that different well-being aspects are positively related with character strengths in a sample of medical students at two points in time. This leads to the second domain of positive traits. There are promising results that the application of signature character strengths (ASCS) has positive effects on well-being and health (Peterson and Seligman, 2004; Harzer and Ruch, 2013). The third domain takes the context (e.g., work or study context) into account (Seligman and Csikszentmihalyi, 2000).

In positive psychology the focus is not on health as the absence of illness, but on health as the “presence of the positive” (Ryff, 1995, p. 99). Emphasis is thus placed on nurturing well-being and character strengths. In order to get more information about possibilities to promote the well-being and the working conditions of physicians-to-be, preventing of burnout, fatigue, or other restrictions of well-being is an important task for academic research (Seligman and Csikszentmihalyi, 2000; McClafferty and Brown, 2014).

This analysis emphasizes the first two domains of positive psychology (including well-being and character strengths), in particular as there is a clear research gap in the context of ASCS of medical students and resident physicians. Moreover this analysis takes additional variables of the third domain (hospital vs. study context) into account, distinguishing it from mainstream research. This research is the first to analyze relations between ASCS, well-being and health of medical students in their first year (Study 1) and resident physicians working at two hospitals in Austria (Study 2).

### Well-being and Health in Medical Education

Well-being has been described in the theoretical and empirical literature to have several aspects: On the one hand, there is subjective well-being (SWB), which encompasses the presence of positive emotions, absence of negative emotions, and satisfaction with life as a whole. On the other, there is psychological well-being, which includes aspects such as engagement, relationships, meaning, mastery, and optimism (Su et al., 2014). Well-being and health are a particularly essential issue for people operating in medical education. Mental as well as physical health status is an essential precondition for, and outcome of, a successful educational process. Even though burnout is not limited to the social sector, besides general health aspects, burnout seems to be a relevant issue to medical students and (resident) physicians. There is consensus among most researchers in the field that burnout contains three main dimensions: emotional exhaustion, cynicism/depersonalization, and reduced professional efficacy (Maslach et al., 2001). The construct of emotional exhaustion is a key dimension of burnout, and defined as “a state of losing mental and psychological resources that has an impact on the quantity of mood, such as depleting and draining mental energy” (Nuallaong, 2013). “Burnout is defined as work-related neurasthenia” (p. 102), which is frequently measured by the single burnout component “emotional exhaustion.” According to Maslach et al. (2009), focusing on this key component in lieu of the other two is the current practice in burnout research for the work context. Research has shown that burnout, and especially the key dimension of emotional exhaustion, is related to physical health outcomes (e.g., cardiovascular disease, diabetes, infections) and mental illness (Shirom et al., 2005). There is strong empirical evidence that burnout is an outcome of task- and organization-related working conditions (demands, stressors, and resources) and has further effects on general well-being and health (Schaufeli et al., 2009).

Research has shown well-being, health and burnout to be important issues for medical students and (resident) physicians. Dyrbye et al. (2014) reported very high burnout rates of 56% among medical students, 60% among resident physicians, and 51% among physicians, compared to the general population. According to their representative studies of 2011 and 2012 in the United States, resident physicians had a higher likelihood of burnout, and medical students of depression, compared to the general population and to other courses of studies (Brazeau et al., 2014). In particular, reports of burnout, distress, and depression in samples of German-speaking medical students (e.g., Kohls et al., 2012; Voltmer et al., 2012), resident physicians (e.g., von dem Knesebeck et al., 2010) as well as experienced physicians (e.g., Heinke et al., 2011; Wurm et al., 2016) correspond to findings in the United States.

### Character Strengths and Their Application

The *Job Demands-Resources Model* (Bakker and Demerouti, 2007) is an occupational stress model, which distinguishes between job demands (e.g., time or work pressure, detrimental working conditions) and resources (e.g., autonomy, social support). An accumulation of demands leads to a “health impairment process” including negative consequences like burnout. In contrast, a “motivational process” is activated through resources, which can lead to higher work engagement and performance. The risk of burnout or health problems arising due to potential stressors such as high job demands can be buffered by job resources. Especially in contexts characterized by a rigid organizational hierarchy and by conditions that are difficult to change – like hospitals – it is well worth also taking personal resources of employees into account. Peterson and Seligman (2004) offer a promising concept of personal resources: the ASCS. In 2004 they described 24 human character strengths which are measurable as individual differences via the *Values in Action Inventory of Strengths* (VIA-IS). The character strengths were grouped into six historically based universal human virtues (namely courage, justice, humanity, temperance, transcendence, and wisdom). Peterson and Seligman (2004) assume that every person has about three to seven character strengths, which are typical of the individual. These so-called signature strengths are defined as strengths which fulfill several criteria such as “a sense of ownership and authenticity,” “intrinsic motivation to use the strength” or “a feeling of excitement while displaying it (…)” (Peterson and Seligman, 2004, p. 18).

Whereas the early years of character strengths research studied strengths possession (by analyzing the means of character strengths), in the recent years an additional focus was placed on the applicability of character strengths in specific contexts. Harzer and Ruch (2013) developed the *Applicability of Character Strengths Rating Scales* (ACS-RS) to “measure the extent to which each of the 24 character strengths of the VIA classification is applicable” in work or private life. Harzer and Ruch (2013) made it possible to measure how situational circumstances foster or hinder an individual in behaving in ways relevant to a specific strength. Moreover, the questionnaire enables to distinguish between several contexts, which allows for a more detailed analysis. The applicability of character strengths and especially of signature character strengths is assumed to be correlated with well-being and health by Peterson and Seligman (2004). Studies actually showed associations with a broad range of positive outcomes such as life satisfaction (e.g., Allan and Duffy, 2014; Douglass and Duffy, 2014; Harzer and Ruch, 2016), different well-being aspects (Harzer and Ruch, 2012, 2013), positive experiences at work including academic or job satisfaction (Littman-Ovadia and Steger, 2010; Harzer and Ruch, 2013; Allan and Duffy, 2014; Lavy and Littman-Ovadia, 2017), and employee engagement (Crabb, 2011; Lavy and Littman-Ovadia, 2017). Studies analyzing character strengths also found positive correlates with healthier work-related behaviors (Gander et al., 2012) and self-evaluated health and physical health in terms of cardio-respiratory fitness, strength, flexibility, and coordination (Proyer et al., 2013), which is congruent with Peterson and Seligman’s theoretical assumption that characters strengths are related to (mental and physical) health (Peterson and Seligman, 2004; Peterson and Park, 2011).

So far, there has rarely been research on character strengths and their applicability in the context of medical education. Ruiz-Aranda et al. (2014) found a relationship between social intelligence (which is also a character strength according to Peterson and Seligman, 2004) and SWB of female student health professionals in a 3 months follow-up. After 12 weeks, students with higher social intelligence reported lower stress levels, higher life satisfaction and happiness. The relationship between social intelligence and well-being was mediated by perceived stress. This shows that high social intelligence increases well-being by reducing the experience of stress. In conclusion, the authors recommend the inclusion of training programs to foster social intelligence in medical curriculums. Some medical universities are already implementing positive psychological interventions, fostering study/work engagement, and taking steps to prevent burnout (Pedrals et al., 2010). Moreover, research has shown that identifying and encouraging students’ strengths and talents is positively correlated with various well-being aspects (Schreiner et al., 2008). Considering the particular stressors and challenges faced by individuals in the health care profession, resources like the ASCS are expected to contribute to preventing burnout, and moreover may promote general well-being and health (e.g., Harzer and Ruch, 2013). Lavy and Littman-Ovadia (2017) assume that the broaden-and-build theory of Fredrickson (2001) works as an underlying mechanism. On this view, positive emotions may be responsible for the positive effects of the application of character strengths on desirable (work-related) outcomes (e.g., Lavy and Littman-Ovadia, 2017). The other way round negative emotions or states (e.g., stress or emotional exhaustion) are also supposed to be negatively related with well-being and health. Similar to perceived stress (Ruiz-Aranda et al., 2014) emotional exhaustion may also have an indirect effect on the links between ASCS, well-being and health, which is the main focus of the present analysis.

### Aims and Hypotheses of the Present Two Studies

The alarming results of impaired health and well-being of health professionals show the need to address the issues surrounding the well-being, health and risk for burnout of medical students and physicians. The promotion of career satisfaction and the prevention of burnout, especially in the beginning of the career, should be aspired to (Dyrbye et al., 2013). The presented theoretical background suggests that ASCS may influence well-being, mental health, and physical health directly as well as indirectly, by way of decreasing burnout symptoms. Taking work and organizational psychology models into account, the specific context (study vs. hospital) was expected to make a difference for whether circumstances would facilitate or hinder ASCS. We conducted two studies to examine the research questions outlined in the following in two different samples: (1) medical students in their first year of studies and (2) resident physicians working in hospitals.

Firstly, character strengths matter in the working context as previous studies have shown (e.g., Gander et al., 2012; Harzer and Ruch, 2013). In the first study, we analyzed the potential positive effects of ASCS in relation to well-being, mental health, and physical health of medical students in their first year of study. We hypothesized that ASCS would be positively linked to well-being (Hypothesis 1a), mental health (Hypothesis 2a), and physical health (Hypothesis 3a). Secondly, referring to the *Job Demands-Resources model* (Bakker and Demerouti, 2007) ASCS could be seen as individual resource in the working context, which may positively impact work engagement and counteract burnout (low ASCS increases the risk for burnout) in a motivational process (e.g., Schaufeli et al., 2009). Research showed evidence for the effects of burnout on general well-being and mental health (Shirom et al., 2005; Cole et al., 2012). Based on this theoretical background, we hypothesized that the relations between ASCS and well-being, mental health, and physical health all are mediated by emotional exhaustion (Hypothesis 4a, 5a, 6a).

In the second study, we aimed to replicate the hypotheses as depicted above in a sample of resident physicians working in hospitals. Again, we expected direct positive relations between ASCS and well-being (Hypothesis 1b), mental health (Hypothesis 2b), and physical health (Hypothesis 3b), as well as indirect effects via emotional exhaustion (Hypothesis 4b, 5b, 6b).

Additionally, following Harzer and Ruch (2013), we expected the specific context and stage of education (first year of medical studies vs. residency) and the different kinds of work (studying and learning vs. working in medical care) to make a difference for ASCS, well-being and health. We hypothesized that medical students have more opportunities to use their character strengths compared to resident physicians working in hospitals (Hypothesis 7) and therefore also have higher levels of well-being (Hypothesis 8), mental health (Hypothesis 9), and physical health (Hypothesis 10).

## Study 1

Study 1 was designed to examine Hypotheses 1a, 2a, 3a, 4a, 5a, and 6a in a sample of medical students. ASCS was investigated in relation to well-being, mental, and physical health. Furthermore, investigating emotional exhaustion as a possible mediator was a central focus of the study.

### Method

#### Participants and Procedure

We recruited three samples of medical students in their first year of studies (January and February, 2015, 2016, and 2017) for an online survey and sent 1488 recruitment emails and two reminders. Incentives offered were direct feedback on participants’ individual five signature character strengths, medical education credits, and a raffle of medical books and vouchers. The response rate was 32% (*N* = 480). We found no significant differences between the three measurement times regarding the dependent variables. We collected complete data of 387 medical students at an Austrian medical university. Sixty-four percent were female with a mean age of 20.7 years (*SD* = 2.5). Most were Austrian (54%) nationals and single (67.4%). The students spent an average of 39 h per week studying (*SD* = 14.9 h, range 2–85). Twenty-four percent reported a secondary employment with an average of 8.7 h per week (*SD* = 4.4 h, range 2–30 h). Detailed demographics can be found in **Table 1**.

**Table 1:** General demographic information (Studies 1 and 2).

	Demographics	Study 1: medical students (%) (*N* = 387)	Study 2: resident physicians (%) (*N* = 136)
Gender	Female	63.6	65.4
	Male	36.4	34.6
Age	17–20	63.3	0
	21–30	35.9	45.6
	31–40	0.8	47.8
	40+	0	6.6
	Mean (*SD*)	20.7 (2.5)	32.0 (4.8)
Nationality	Austrian	54.9	71.3
	German	22.2	14.0
	Italian	18.6	11.0
	Others	5.2	3.7
Marital status	Single	67.4	24.3
	Partnership/married	32.6	75.7
Living	Alone	23.0	26.5
	With partner/family	7.5	65.4
	Flat-sharing community	45.3	5.1
	Family of origin	15.2	2.9
(Desired/current)^a^ Discipline	Surgery	12.1	7.4
	Pediatrics	9.0	4.4
	Neurology	3.6	1.5
	General medicine	6.5	0
	Psychiatry	1.6	8.1
	Radiology	0.5	8.1
	Anesthesia	4.9	20.6
	Internal Medicine	2.8	16.2
	Others	22.0	6.6
	Still unclear	37.0	0


*^a^Desired discipline of medical students, current discipline where resident physicians are working in.*

#### Instruments

The *Comprehensive Inventory of Thriving (CIT)* (Su et al., 2014) was used to measure well-being. It comprises 54 items rated on a five-point Likert scale and ranges from 1 = *strongly disagree* to 5 = *strongly agree*. The CIT measures subjective and psychological well-being including items measuring positive emotions, relationships, engagement, autonomy, meaning in life, mastery, and optimism. Sample items are “My life is going well” or “I am achieving most of my goals.” The German translation has shown to be valid and reliable (Hausler et al., 2017a). The CIT has an excellent internal consistency with α = 0.94 for SWB and α = 0.91 for PWB in the sample of freshmen.

The German version of the *Short Form Health Survey (RAND-SF-12)* (Bullinger and Kirchberger, 1998) measures mental and physical health composite scores (MCS and PCS) within the last 4 weeks with 12 items. The composite scores are computed by regression with different weights. Sample items are “In the past 4 weeks, did you accomplish less than you would like as a result of an emotional problem, such as feeling depressed or anxious?” (MCS) or “Thinking about the past 4 weeks, have you accomplished less than you would like as a result of your physical health?” (PCS). The test-retest reliability for the subscales were 0.89 (PCS) and 0.76 (MCS) (Ware et al., 1996).

The German 120 item version of the *Values in Action Inventory of Strengths (VIA)* (Via Institute on Character, 2014) was used to measure individual character strengths. The scale ranges from 1 = *strongly disagree* to 5 = *strongly agree*. Psychometric properties in our study were similar to the original 240 items version with Cronbach’s α ranging from α = 0.65 (*hope)* to α = 0.89 (*spirituality*) for the short version (Littman-Ovadia, 2015). Sample items are “I have many interests” (curiosity) or “I feel thankful for what I have received in life” (gratitude). Several studies have shown the factorial validity of the original version (e.g., Harzer and Ruch, 2013, 2015).

The German *Applicability of Character Strengths Rating Scales* (ASCS-RS) (Harzer and Ruch, 2013) were used to evaluate the applicability of individual character strengths in their studies. Harzer and Ruch describe that the combination of the VIA-120 and the ACS-RS “allows for an operationalization of the strengths-related congruence between an individual and the situational circumstances at work. This congruence is proportional to the extent to which a job allows for the application of one’s signature strengths” (Harzer and Ruch, 2012, pp. 5–6).

According to Peterson and Seligman (2004), every person has about three to seven character strengths, which are typical of the individual. Based on this assumption, applicability in student life was measured for each participant’s five highest character strengths identified within the VIA survey. Each of these individual character strengths was addressed with four questions in the ASCS scale, rated on a five-point scale from 1 = *never* to 5 = *(almost) always*. The items inquire whether the strength is encouraged, helps the individual, is important, and to which extent it is possible to act according to the strength in the respective context. A good internal consistency of the ASCS-RS with α = 0.84 was found in the present study.

Emotional exhaustion, the key dimension of burnout, was measured using the adapted and modified German student version of the Maslach-Burnout-Inventory (MBI-SS-GV) by Gumz et al. (2013). It consists of 21 items with a six-point scale from 1 = *never* to 7 = *very often*, and showed good reliability (α = 0.87). A sample item is “I feel burnt out from my work.”

#### Data Analysis

Data were analyzed using IBM SPSS Statistics 22. Frequencies, means, and SD were used to describe the sample characteristics. Correlations and regressions including mediations were designated. The latter were calculated with the SPSS macro PROCESS by Preacher and Hayes (Hayes, 2013). The mediation analyses were based on bootstrapping (10,000 bootstrap samples) using 95% confidence intervals. The macro allows calculating and testing the direct effect (regression controlled for the mediator), the total effect (regression without including the mediator), and the indirect effect. The latter is significant when the 95% CI does not include 0. Sex, age, and marital status were included as controls.

### Results

First, in the preliminary analyses several outliers (ACS-RS: *N* = 2; PWB: *N* = 1; SWB: *N* = 1; PCS: *N* = 3) could be identified and were deleted (critical z-value of ±3.29) resulting in a final N = 387. The PWB and ASCS-RS scores were normally distributed [Kolmogorov–Smirnoff Test (KS-test), histogram]. SWB, PCS, MCS, and MBI-EE were non-normally distributed with the following values of skewness (*SE* = 0.12) and kurtosis (*SE* = 0.25) for SWB (-0.71; 0.44), PCS (-1.04; 1.05), MCS (-0.30; -1.05), and MBI-EE (0.44; 0.194). This shows that students were relatively physically and mentally healthy, reported little emotional exhaustion and high SWB.

Second, means, SD, and internal consistencies of the measures were computed. Third, relations of the variables with demographics (age, sex, marital status) were calculated, as proposed in several studies (e.g., Harzer and Ruch, 2012; Wagner and Ruch, 2015). Men showed a statistically higher mental health than women (*r* = 0.17; *p* < 0.001), older students had lower scores in ASCS (*r* = -0.12; *p* = 0.020), and marital status had no statistical effect. All subsequent analyses were controlled for the demographics of sex, age, and marital status.

Partial correlation analyses showed significant relationships of ASCS with all variables except physical health. Effect sizes were moderate for well-being and small for mental health. Hypothesis 1a (well-being) and 2a (mental health) were confirmed; Hypothesis 3a (physical health) was not (**Table 2**).

**Table 2:** Reliability, descriptives, and partial correlations of well-being, application of signature character strengths, health, and emotional exhaustion of medical students (Study 1) and resident physicians (Study 2).

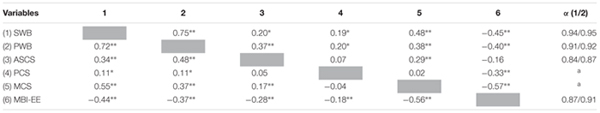

**N* = 387 (Study 1, left, down the line); *N* = 136 (Study 2, right, above the line); controlled for sex, age, and marital status; SWB, subjective well-being; PWB, psychological well-being; ASCS, application of signature character strengths; PCS, SF-12 physicial health score; MCS, SF-12 mental health score; MBI-EE, emotional exhaustion; α, Cronbach’s alpha; Pearson’s correlation coefficients: correlations of < 0.10 = none, 0.10–0.30 = weak, 0.30–0.49 = moderate, ≥50 = high; ^∗^statistical significance level of *p* ≤ 0.05, ^∗∗^statistical significance level of *p* < 0.01; ^a^The SF-12 composite scores are computed by regression with different weights, therefore Cronbach’s alpha cannot be calculated.*

**FIGURE 1:**
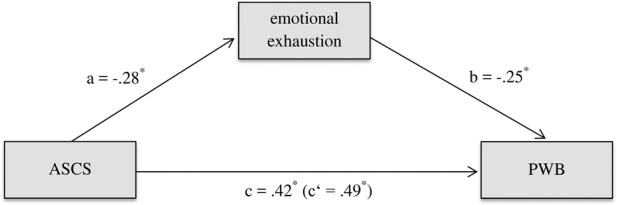
The mediating role of emotional exhaustion in explaining the relation between the application of signature character strengths and psychological well-being of medical students (Study 1). Medical students (*N* = 387); controlled for sex, age, and marital status; the paths are unstandardized; ASCS = application of signature character strengths at work; PWB = psychological well-being; *a* = direct effect of ASCS on mediator; *b* = direct effect of mediator on PWB; *c* = total effect of ASCS on PWB; *c*′ = direct effect of ASCS on PWB ^∗^*p* < 0.05.

Based on these results, *emotional exhaustion* was analyzed as a possible mediator (**Table 3**). Results showed a significant indirect effect of *emotional exhaustion* on SWB and PWB (**Figure 1**). Hypothesis 4a was thus confirmed for both well-being aspects.

**Table 3:** Mediation analyses of medical students (Study 1).

Paths	*a*	*b*	*c*	*c’*	*a^∗^b*	95% CI of *a^∗^b*	*SE*	*R*^2^
ASCS → EE → SWB	-0.28^∗∗^	-0.36^∗∗^	0.34^∗∗^	0.24^∗∗^	0.10^∗^	[0.06, 0.16]	0.025	0.26
ASCS → EE → PWB	-0.28^∗∗^	-0.25^∗∗^	0.49^∗∗^	0.42^∗∗^	0.07^∗^	[0.04, 0.12]	0.019	0.30
ASCS → EE → MCS	-0.28^∗∗^	-0.54^∗∗^	0.17^∗∗^	0.02	0.15^∗^	[0.09, 0.22]	0.031	0.33
ASCS → EE → PCS	-0.28^∗∗^	-0.16^∗∗^	0.05	0.00	0.05^∗^	[0.02, 0.09]	0.018	0.04


**N* = 387; controlled for sex, age, and marital status; the displayed effects are standardized; ASCS, application of signature character strengths at work; SWB, subjective well-being; PWB, psychological well-being; MCS, mental health score; PCS, physical health score; EE, MBI emotional exhaustion; ^∗^statistical significance level of *p* ≤ 0.05, ^∗∗^statistical significance level of *p* < 0.01, the indirect effect is significant (^∗^) when the 95% CI does not include 0; *SE*, bootstrap regression standard error; *R*^2^, variance accounted for; *c*, total effect; *c’*, direct effect; *a^∗^b*, indirect effect.*

Moreover, a significant indirect effect of e*motional exhaustion* on mental health was found (**Table 3**). Therefore, Hypotheses 5a was also confirmed.

No impact of ASCS on *physical health* was found in the previous correlational analyses. Nevertheless, a significant indirect effect through *emotional exhaustion* was found. Interpretation was based on Hayes’ (2013) statement that interpreting a significant effect is reasonable even if there is no significant total effect. Thus, Hypothesis 6a was also confirmed.

#### Summary of Results of Study 1

Results of Study 1 showed that ASCS was significantly related to the well-being and mental health but not the physical health of medical students. We found significant indirect effects of e*motional exhaustion* on all three direct links.

## Study 2

Aim of Study 2 was to replicate the findings of Study 1 in a sample of resident physicians working in hospitals. A further aim was to compare the results and the initial levels of well-being, ASCS, mental and physical health between the two samples to take the differences between the study and work context into account.

### Method

#### Participants and Procedure

At three time points (2015–2016) a total of 1266 recruitment emails and two reminders were sent to resident physicians. Resident physicians are physicians in training, having worked in a hospital between three and a half and six years. They have finished medical school, have a medical degree and are supervised during their work at the hospital. Out of 236 respondents (19%), datasets for 157 participants were complete. The resident physicians were 2 months’ minimum on the job. Nineteen had already finished their residency and were therefore excluded. Data was gathered through an online survey. Incentives offered were direct feedback of participants’ individual five signature character strengths, and vouchers for brunch. Again, no significant differences between the three measurement times regarding the dependent variables were found.

Complete data of 136 resident physicians were collected in two hospitals in Austria (**Table 1**). 65.4% of the resident physicians were female, with a mean age of 32.0 years (*SD* = 4.8, range: 24–49). Most were Austrian (71.3%) nationals, most were in a partnership (75.7%), and 25% had children. Most of the resident physicians were living with their partner or family (65.4%). The resident physicians reported an average contractual working time of 39.94 h (*SD* = 7.94) and an actual working time of 48.76 h (*SD* = 10.00) per week.

#### Instruments

Again, well-being was measured with the CIT (Su et al., 2014), health was measured with the RAND-SF-12 (Bullinger and Kirchberger, 1998), character strength possession with the 120 items of the VIA-IS (Via Institute on Character, 2014) and the applicability of each participant’s signature character strengths with the ACS-RS (Harzer and Ruch, 2013). In contrast to study 1, burnout was measured with scales specifically addressing the work context, again focusing on the key component of burnout: Emotional exhaustion was measured with the adapted and modified German version of the Maslach-Burnout-Inventory for the work context of human services from 1 = *never* to 6 = *very often* (MBI-D; Büssing and Perrar, 1992). The Cronbach’s Alphas of the 24 VIA-120 scales ranged from α = 0.60 (*authenticity)* to α = 0.90 (*spirituality*). All other scales had satisfying reliabilities throughout (**Table 2**).

#### Data Analysis

Data were analyzed using IBM SPSS Statistics 22. Frequencies, means (M), and SD were used to describe the sample characteristics. Analyses of covariance (ANCOVAs) were used to compare the means between the samples. Correlations and regressions including mediators and covariates (sex, age, and marital status) were examined. The latter were calculated with the SPSS macro PROCESS by Preacher and Hayes (Hayes, 2013).

### Results

In the preliminary analyses two outliers (SWB: *N* = 1; PCS: *N* = 1) could be identified (critical *z*-value of ±3.29) and were deleted. The final dataset consisted of *N* = 136 resident physicians. MBI-EE, ASCS, and PWB were normally distributed (KS-test, histogram). SWB, PCS, and MCS were non-normally distributed with the following values of skewness (*SE* = 0.21) and kurtosis (*SE* = 0.41) for SWB (-0.65; 0.38), PCS (-1.28; 1.05), and MCS (-0.70; -0.44). This shows that the resident physicians, too, were relatively healthy and had high SWB. Means, SD, and reliability of the instruments were computed. The means at t1 and t2 were then compared for statistical differences (ANCOVA). Lastly, possible covariates were analyzed. Sex, age, and marital status had no statistical effect. As in Study 1 all subsequent analyses were again controlled for demographics.

Correlation analyses showed that ASCS was significantly (*p* < 0.05) correlated with SWB, PWB, and mental health but not with physical health or burnout (**Table 2**). Effect sizes were moderate for PWB and small for SWB and mental health. Results confirmed Hypotheses 1b and 2b, but not 3b.

*Emotional exhaustion* was found to have a significant indirect effect on SWB, PWB, and physical health. No significant indirect effect on *mental health* was identified. Thus, Hypotheses 4b (for both well-being aspects) and 6b were confirmed; Hypothesis 5b was not (**Table 4**).

**Table 4:** Mediation analyses of resident physicians (Study 2).

Paths	*a*	*b*	*c*	*c*’	*a^∗^b*	95% CI of *a^∗^b*	*SE*	*R*^2^
ASCS → EE → SWB	-0.16	-0.41^∗∗^	0.19^∗^	0.13	0.07^∗^	[0.003, 0.16]	0.038	0.24
ASCS → EE → PWB	-0.16	-0.34^∗∗^	0.37^∗∗^	0.32^∗∗^	0.06^∗^	[0.004, 0.13]	0.031	0.28
ASCS → EE → MCS	-0.16	-0.53^∗∗^	0.30^∗∗^	0.21^∗∗^	0.09	[-0.003, 0.20]	0.052	0.37
ASCS → EE → PCS	-0.16	-0.30^∗∗^	0.07	0.02	0.05^∗^	[0.003, 0.13]	0.031	0.14


**N* = 92; controlled for sex, age, and marital status; the displayed effects are standardized; ASCS, application of signature character strengths at work; SWB, subjective well-being; PWB, psychological well-being; MCS, mental health score; PCS, physical health score; EE, MBI emotional exhaustion; ^∗^statistical significance level of *p* ≤ 0.05, ^∗∗^statistical significance level of *p* < 0.01, the indirect effect is significant (^∗^) when the 95% CI does not include 0; *SE*, bootstrap regression standard error; *R*^2^, variance accounted for; c, total effect; c’, direct effect; a^∗^b, indirect effect.*

### Summary of Results of Study 2

Results of Study 2 showed that ASCS was significantly related to well-being and mental health, but not to physical health of resident physicians. We identified significant indirect effects of *emotional exhaustion* on the links between ASCS and well-being and ASCS and physical health. We found no significant indirect effect on the ASCS-mental health link. All indirect effects were relatively small in Study 2.

## Comparison of Study 1 and 2

Several ANCOVAs were conducted to examine the differences between the two samples concerning their ASCS, well-being, and health. The ANCOVAs were computed with sample (sample 1 vs. sample 2) as between-subject factor, demographics (sex, age, and marital status) as covariates, and SWB, PWB, ASCS, MCS, and PCS as dependent variables. Results indicated significant differences between the two samples in the dependent variables of ASCS and mental health after controlling for demographics (**Table 5**). Thus, results showed that resident physicians had significantly higher means of ASCS and mental health compared with medical students. Both samples had similar means of SWB, PWB, and physical health.

**Table 5:** Descriptive results and summary of ANCOVAs (Study 2).

	Means (*SD*) of sample 1	Means (*SD*) of sample 2	*F*[1,518]	*p*	η2
SWB	4.05 (0.68)	3.93 (0.70)	0.381	0.538	0.001
PWB	4.03 (0.39)	3.86 (0.39)	0.848	0.358	0.002
ASCS	3.82 (0.49)	3.86 (0.52)	7.258	0.007	0.014
MCS	41.68 (10.38)	44.53 (9.77)	5.673	0.018	0.011
PCS	55.92 (5.37)	54.92 (6.37)	3.07	0.580	0.001
MBI-EE	2.76 (1.11)	2.40 (0.99)	^a^	^a^	^a^
*N*	387	136			


*Sample 1, medical students; sample 2, resident physicians; controlled for sex, age, and marital status; ^∗^statistical significance level of *p* ≤0.05, ^∗∗^statistical significance level of *p* < 0.001; ^a^the two versions of the questionnaires are not comparable with each other. SWB, subjective well-being; PWB, psychological well-being; ASCS, application of signature character strengths at work; MCS, mental health score; PCS, physical health score; MBI-EE, MBI emotional exhaustion.*

In addition, we compared the differences between the correlation coefficients (ASCS, well-being, and health scores) between Studies 1 and 2 with each other. We found no significant differences (Fisher’s *z*-transformation). In conclusion, Hypotheses 7, 8, 9, and 10 were not confirmed.

Results of both studies showed that ASCS was directly linked to well-being and mental health, but not to physical health. Moreover, the mediation analyses showed significant indirect effects of emotional exhaustion on the links between ASCS, well-being and physical health. The results regarding ASCS and mental health were inconsistent (**Tables 3**, **4**).

## General Discussion

In this analysis, we investigated the links between the applicability of signature character strengths at study or work and well-being, mental and physical health. Burnout was taken into account as a possible mediator. In summary, we found ASCS to be positively related to well-being and health. Future longitudinal investigations may reveal whether ASCS could be relevant for burnout prevention interventions for medical students and resident physicians.

In this analysis, as expected, the more ASCS participants reported in their studies (Study 1) and work (Study 2), the higher their levels of well-being and mental health. We found significant indirect effects between ASCS and well-being through *emotional exhaustion* in both studies. In line with Peterson and Seligman (2004), *physical health* was significantly correlated with well-being. In contrast to their assumptions, we found no significant correlations with ASCS. Both samples showed an average physical health status with limited variance, which may explain these results. However, the assessed sample is a specific one. In the general population, with more heterogeneity and variance in physical health, different results may be found. Moreover, a significant indirect effect of *emotional exhaustion* was found on the link between physical health and ASCS in Study 2. The indirect effect of *emotional exhaustion* on the links between ASCS and physical health (both studies) were relatively weak, perhaps due to the relatively small variance of physical health in these specific samples.

The results were inconsistent regarding the significance of the indirect effect of *emotional exhaustion* on the link between ASCS and mental health. Whereas the link was clearly significant in Study 1, the effect was not significant in Study 2, albeit approaching nearly significance with a confidence interval near zero. This inconsistency seems to be mainly due to the smaller contribution of ASCS to emotional exhaustion in resident physicians than in medical students. Moreover, all indirect effects in Study 2 were relatively small. Other factors such as working conditions, stressors or other mental health restrictions may be more central for (the development of) burnout. Glaser et al. (2015) developed and analyzed a model including learning demands, resources, and stressors in the working context. Based on previous research and due to their study results they assume that “challenging work tasks and learning requirements may be detrimental to health if this improvement is accompanied by work overload” (p. 44). This may be an explanation for the differences in Studies 1 and 2.

In a further step, we compared the levels of well-being, ASCS, mental and physical health between the two samples across the studies to take into account differences between the study and work context. The results showed moderately higher means in ASCS and mental health for resident physicians compared to resident medical students, while all other variables were similar between both samples, with no statistically significant differences. Possible reasons for this could be differences in workflow or task related issues. Medical students may have lesser opportunities to use their character strengths during their studies (e.g., preparing for exams) compared to the resident physicians, who are working in their field of expertise. Further study designs should take these possible causes for the observed differences in well-being into account.

Harzer and Ruch have stated in 2013 that there is a lack of studies analyzing the specific role of character strengths in the context of work. In particular, they have recommended an investigation of specific jobs to look for the most appropriate character strengths and their application in the context of those jobs. We are not aware of any previous studies that have analyzed character strengths applicability in a sample of medical students and resident physicians, despite the particular risk for burnout, health problems, and reduced well-being for these groups in particular. Our research is a first step toward an understanding of whether fostering the application of character strengths may be a promising approach in medical education. Harzer and Ruch (2013) further emphasized the importance of opportunities to use one’s individual character strengths at work in order to promote job satisfaction and engagement as well as productivity. Moreover, Gander et al. (2012) and Proyer et al. (2013) reported on positive correlates with healthier work-related behaviors and physical health, emphasizing the potential of character strengths interventions for health outcomes. In light of the predominant strains on medical students and practitioners, this is an important potential resource to consider. In light of this, it is especially important to investigate possible benefits of character strengths in the setting of medical education. Fostering ASCS as part of the education process may have positive effects on well-being, job (and presumably life) satisfaction, and work engagement. Peterson and Park (2006) described character strengths as a “neglected but critically important resource for organizations.” Harzer and Ruch (2013) also stated that congruence between the character strengths of a person and work demands play an important role. That means it would be a sustainable win–win situation for both the person and the organization if the daily routine and the demands of medical study or later work as a (resident) physician were congruent with the respective individual’s personal character strengths. Veenhoven (2008) even describes a direct possibility of influencing public health by strengthening individual life-abilities. Teaching or learning character strengths could be a way of doing so.

### Limitations and Future Research

Limitations of the present study were the different sample sizes and the cross-sectional study design using samples from only one medical university and hospitals from one state and country. Further research might aim to examine the links found in this study in the general population, at different workplaces and situations, or even in other life domains (e.g., private life: leisure time, in special relationships, on holidays). Another limitation was that some of the indirect effects found were relatively small or not significant. A replication of this study design may potentially strengthen results. For all mediations it has to be considered that the cross-sectional design does not allow for any causal interpretations.

Taking into account the described impact of ASCS, future research should address its potential of being a general predictor in longitudinal designs. Working climate or working conditions (e.g., stimulating job demands and resources) are potential influencing factors. In addition, the connection between ASCS and work-related well-being should be investigated in more detail: Are individuals with higher ASCS at work likely to feel competent and self-confident more often in fulfilling their individual needs and, consequently, is their generalized expectation of self-efficacy (Bandura, 1977) higher? Van Woerkom et al. (2016), for example, identified associations between strengths use and work engagement and self-efficacy of employees at work. Furthermore, in South Africa work engagement was found to be positively associated with perceived organizational support for strengths use (Botha and Mostert, 2014). Studies taking the application of character strengths in different cultures into account will be a strong enrichment for character strengths research. A very recent approach of Littman-Ovadia et al. (2016) showed that using signature strengths and using happiness strengths (hope, love, gratitude, curiosity, zest) both proved beneficial for different aspects of work. In contrast to the present study, signature strengths were associated with aspects of functioning at work (e.g., performance) but not with emotional-psychological aspects (e.g., work engagement) and vice versa for happiness strengths. The authors concluded that universal happiness strengths at work may evoke more positive emotions than using one’s individual signature strengths at work. The effects of using signature strengths seem to be related to additional mechanisms, which could be even more central than positive emotions. Littman-Ovadia et al. (2016) stressed the potential benefits for individuals and organizations of using a variety of strengths in the working context. They further emphasized the importance of examining additional mechanisms connected with the effects of signature strengths use. They concluded that beyond the effects of signature strengths use, there may be an additional benefit to using one’s lowest-ranked strengths, an aspect our study did not investigate. Future study designs may throw light on possible positive effects of applying one’s signature strengths, other clusters of strengths, and using strengths in general in different contexts (e.g., Huber et al., 2017).

A preventative approach including the concept of character strengths could help enhance well-being and health, and reduce the risk of burnout, for medical students and people in the medical field. Future (research) efforts to improve medical curricula may include the identification of character strengths that generally tend to be useful in the context of medical education. As a result, it could be investigated how these strengths may be promoted in medical students from the outset. Moreover, creating an awareness of (signature) character strengths (for the students themselves and for others) should be tested for its positive impact on students’ well-being and mental health as well as work engagement and zest for their profession. Teaching and fostering individual character strengths (e.g., through strength workshops and strength-building activities) in the educational and the working context could be a first step toward implementing this research in practice. Fostering character strengths through changing working and studying conditions toward a positive institution may be expected to increase well-being and health across the entire institution. Moreover, the potential advantages of a fit between individual character strengths and workplaces involving or fostering personal resources should specifically become a stronger focus in future research.

## Conclusion

The results of these studies have the following implications:

Our analysis has shown that the ASCS is positively linked to well-being and mental health. Moreover, people who are able to use their signature character strengths at study or work are less likely to suffer from burnout (or vice versa).

Our findings underline the potential of fostering the ASCS. As a result we recommend the experimental testing of an implementation of character strengths training for people studying or working in the context of medical education. Future research should analyze possible improvements of medical curricula by including the concept of character strengths in order to enhance the well-being and health of medical students and people operating in the medical field.

## Ethics Statement

This study was carried out in accordance with the recommendations of “The Board for Ethical Questions in Science of the University of Innsbruck” with written informed consent from all subjects. All subjects gave written informed consent in accordance with the Declaration of Helsinki. The protocol was approved by the “The Board for Ethical Questions in Science of the University of Innsbruck.”

## Author Contributions

All authors were substantially involved in the planning of the study, the interpretation of data and revision of the article. MH and CS handled the data collection. MH conducted the data analysis and the writing of the article.

## Conflict of Interest Statement

The authors declare that the research was conducted in the absence of any commercial or financial relationships that could be construed as a potential conflict of interest.
